# Proteostasis and neurodegeneration: a closer look at autophagy in Alzheimer's disease

**DOI:** 10.3389/fnagi.2023.1281338

**Published:** 2023-11-02

**Authors:** Haleh Barmaki, Alireza Nourazarian, Fatemeh Khaki-Khatibi

**Affiliations:** ^1^Department of Biochemistry and Clinical Laboratories, Faculty of Medicine, Tabriz University of Medical Sciences, Tabriz, Iran; ^2^Department of Basic Medical Sciences, Khoy University of Medical Sciences, Khoy, Iran

**Keywords:** Alzheimer's disease, proteostasis, autophagy, neurodegeneration, oxidative stress

## Abstract

Alzheimer's disease (AD) is characterized by the accumulation of misfolded amyloid-beta and tau proteins. Autophagy acts as a proteostasis process to remove protein clumps, although it progressively weakens with aging and AD, thus facilitating the accumulation of toxic proteins and causing neurodegeneration. This review examines the impact of impaired autophagy on the progression of AD disease pathology. Under normal circumstances, autophagy removes abnormal proteins and damaged organelles, but any dysfunction in this process can lead to the exacerbation of amyloid and tau pathology, particularly in AD. There is increasing attention to therapeutic tactics to revitalize autophagy, including reduced caloric intake, autophagy-stimulating drugs, and genetic therapy. However, the translation of these strategies into clinical practice faces several hurdles. In summary, this review integrates the understanding of the intricate role of autophagy dysfunction in Alzheimer's disease progression and reinforces the promising prospects of autophagy as a beneficial target for treatments to modify the course of Alzheimer's disease.

## 1. Introduction

Alzheimer's disease (AD) is a progressive neurodegenerative disorder that is the leading cause of dementia in the elderly population. This condition contributes to ~70% of the estimated 50 million cases of dementia worldwide (Morato et al., 2022; Alzheimer's Association, 2023). The hallmark pathological features of AD include the accumulation of amyloid beta (Aβ) plaques and the presence of neurofibrillary tangles of hyperphosphorylated tau protein in the brain (Hampel et al., 2021). These manifestations contribute to synaptic dysfunction and neuronal decline. The origins of sporadic or late-onset AD, which accounts for more than 95% of cases, remain largely unknown. However, they are likely influenced by a multifaceted interplay of genetic, epigenetic, and environmental factors (Gcwensa et al., 2021). Pathways implicated in the development of AD include deficiencies in Aβ clearance, tau-related pathology, neuroinflammation, mitochondrial dysfunction, and impaired proteostasis (Gadhave et al., 2020).

Proteostasis involves the regulatory processes that maintain the balance of the proteome through functions such as protein synthesis, folding, degradation, and catabolism, mediated by pathways such as the ubiquitin–proteasome system and autophagy. In situations of proteostatic imbalance, the heat shock response and the unfolded protein response step in to correct the situation, but their restorative efficiency declines with age (Lei et al., 2021; Sarkar and Nazir, 2022). The inability to restore proteostasis leads to proteotoxic stress and eventual cell death. Neurodegenerative disorders, such as AD, are characterized by ubiquitous proteostasis perturbations. However, whether these perturbations trigger or result from neurodegeneration remains a subject of ongoing debate (Hohn et al., 2020; Ruz et al., 2020).

Autophagy is an intracellular catabolic pathway involving the lysosomal degradation of cellular components from the cytoplasm. Autophagy begins with the formation of double-membrane vesicles called autophagosomes, which engulf materials such as misfolded proteins and damaged organelles. These autophagosomes then fuse with lysosomes to facilitate breakdown and recycling of the contents. Basal autophagy plays a critical role in maintaining cellular integrity by regulating quality control, and can be upregulated in response to stressors. Dysregulation of autophagy has been observed in several neurodegenerative diseases, and also shows declining efficacy over time, which has been implicated in cytotoxic protein accumulation in AD (Hughes et al., 2020; Senos Demarco and Jones, 2020).

In this review, we examine the complex interplay between aberrant proteostasis, autophagy dysfunction, and neuronal death in Alzheimer's disease pathogenesis. We explore proteostasis regulation and molecular details of the autophagy pathway. By evaluating evidence from AD models and human studies, we aim to elucidate this intricate relationship between proteostasis, autophagy, and neurodegeneration in AD, shedding light on mechanisms and revealing new therapeutic avenues.

## 2. Proteostasis and cellular homeostasis

**Figure 1:**
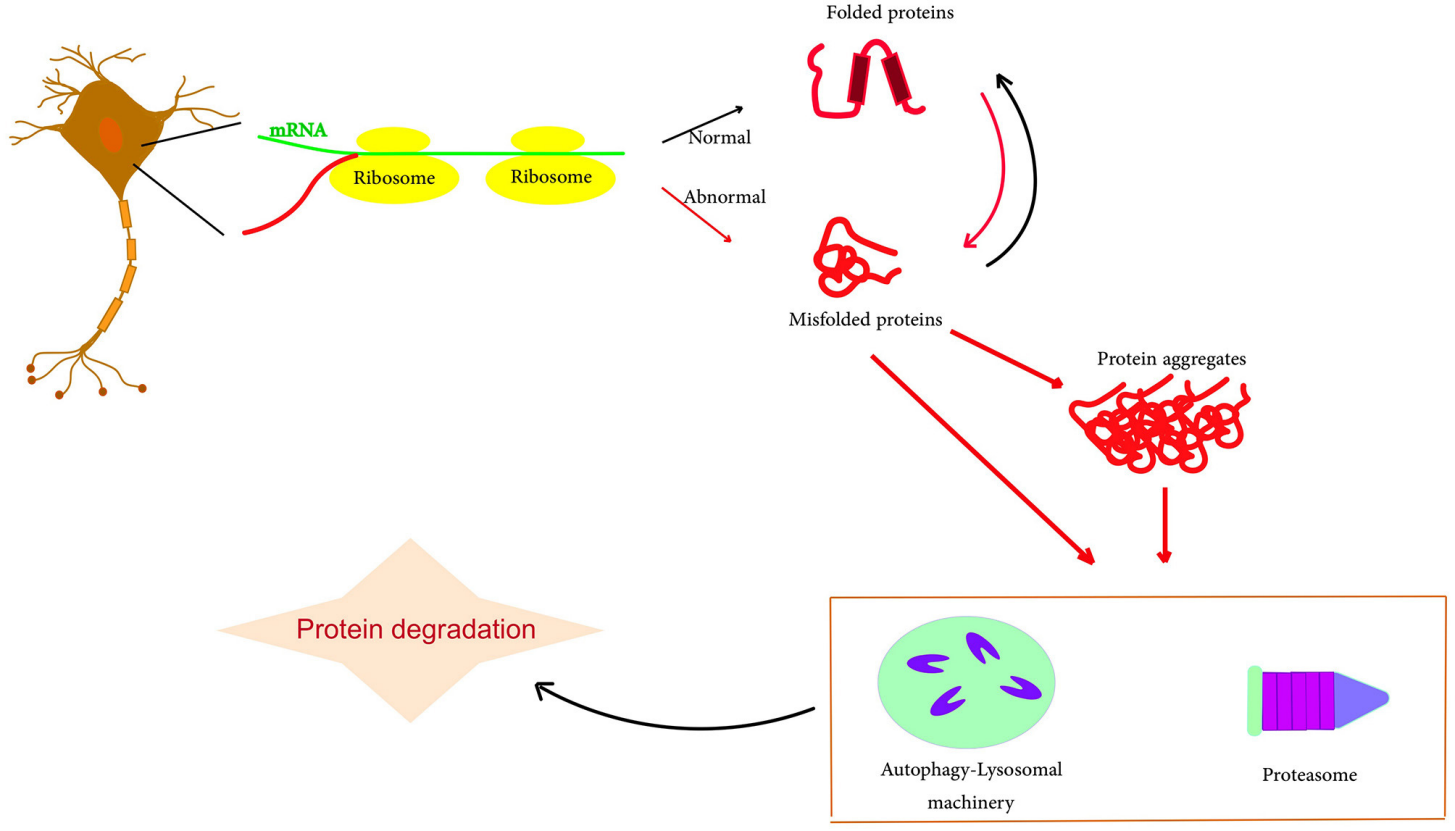
Schematic representation of protein misfolding and aggregation. The accumulation of misfolding proteins, including amyloid-beta and tau, due to an imbalance in protein production. Typically, these abnormal protein clusters are broken down through a process called autophagy or with the support of the cell's proteasome. However, if there is any malfunction in this degradation process, the accumulation of amyloid and tau can potentially escalate, particularly contributing to the progression of AD.

Proteostasis refers to a set of processes that maintain the structure and function of the proteome. It includes mechanisms that regulate protein synthesis, folding, unfolding, and degradation to ensure that proteins achieve the 3D shapes necessary for their biological roles (Sebastian and Shoulders, 2020; Powers and Gierasch, 2021). Disruptions in proteostasis are associated with diseases characterized by protein misfolding and aggregation, such as neurodegenerative disorders (Cortes Sanchon et al., 2021). The key components of proteostasis are molecular chaperones, which are proteins that mediate folding. Chaperones such as Hsp70 and Hsp90 recognize exposed hydrophobic regions on misfolded proteins. They maintain these proteins in a foldable state until proper folding is achieved through ATP hydrolysis and cochaperone action (Frumkin et al., 2014; Saibil, 2021). Notably, chaperones also direct misfolded proteins toward degradation. Aging impairs the effectiveness of the heat shock response, which activates chaperones to deal with proteotoxic stress, leading to proteostatic failure (Morimoto, 2020). In addition, the ubiquitin–proteasome system (UPS) is a major proteolytic pathway that directs the degradation of damaged ubiquitinated proteins. Through “proteasomal degradation”, the UPS removes defective oxidized, mutated, or misfolded proteins, thereby maintaining proteostasis (Fhu and Ali, 2021; Kong et al., 2021). UPS dysfunction leads to protein accumulation, a hallmark of neurodegenerative diseases such as Parkinson's disease, which is associated with reduced UPS function and aggregation of α-synuclein targeted for UPS degradation (Zheng et al., 2016; Al Mamun et al., 2020). In neurodegenerative diseases, protein aggregates accumulate in the neurons, in part due to impaired proteostatic mechanisms (Figure 1; Agius, 2016). Abnormal aggregation into insoluble fibrils can damage membranes, disrupt trafficking, and lead to synaptic dysfunction and neuronal death. Therefore, maintaining neuronal proteostasis is critical to mitigate neurotoxicity. Table 1 shows the components of proteostasis.

**Table 1:** Overview of proteostasis components.

**Category**	**Role in proteostasis**	**Implication in Alzheimer's disease**	**References**
Chaperones	These specific proteins contribute to the correct folding of other proteins and maintain misfolded proteins in a state that allows for possible refolding. They also direct irreversibly misfolded proteins toward the process of degradation.	Dysfunction in the activity of chaperone proteins can lead to the misfolding and subsequent aggregation of proteins such as amyloid-beta, playing a major role in the pathological progression of AD.	Cheng et al., 2018; Izumi, 2019
Proteasomes	Proteasomes play a critical role in degrading aged or damaged proteins, maintaining the balance between protein synthesis and degradation.	Proteasome dysfunction could lead to the accumulation of misfolded proteins and increase the likelihood of protein clumping, a notable feature of AD.	Jones and Tepe, 2019; Hu et al., 2020
Autophagy	Autophagy is the process by which cells remove and degrade superfluous or ineffective components. It promotes cellular wellbeing by recycling cellular components to contribute to essential metabolic processes, thereby supplying the cell with nutrients during periods of nutrient deficiency.	Impaired autophagy can lead to the accumulation of proteins such as amyloid beta and tau. This dysfunction has been implicated in the pathogenesis of AD.	Nieto-Torres and Hansen, 2021; Zhang et al., 2021

In summary, proteostasis regulates processes that manage protein lifecycles to maintain proteome integrity using specialized components such as chaperones, targeted proteolytic mechanisms, and stress responses. Elucidating the dysregulation of neuronal proteostasis in disease may reveal potential areas for therapeutic intervention.

## 3. Autophagy: mechanisms and regulation

Autophagy, a well-conserved lysosomal degradation pathway, performs the vital task of degrading and recycling cellular components (Schaefer and Dikic, 2021). This complex process consists of three primary types: macroautophagy, microautophagy, and chaperone-associated autophagy. Macroautophagy is the process by which cytoplasmic substances are packaged into vesicular structures known as autophagosomes, which then associate with lysosomes to facilitate degradation and recycling of the contents (Schuck, 2020; Wang L. et al., 2022). Microautophagy involves direct engulfment of the cytoplasm into lysosomes via invagination of the lysosomal membrane. The third type, chaperone-mediated autophagy, involves specific proteins called “chaperones” that recognize, guide, and transfer substrate proteins across the lysosomal membrane to the lumen for subsequent degradation (Kaushik and Cuervo, 2018; Schuck, 2020).

Among these types, macroautophagy has been the most thoroughly studied and defined. Effective execution of macroautophagy depends on ATG proteins, which regulate the sequential progression of autophagosomes from initiation to completion (Schuck, 2020; Metur and Klionsky, 2021). These processes are initiated by upstream signaling events that inhibit mTORC1, a nutrient sensor, which then facilitates the activation of the ULK1 kinase complex and the downstream generation of PI3P lipids, which are essential for the recruitment of other ATG proteins (Shimobayashi et al., 2013; Condon and Sabatini, 2019).

Autophagosome formation is highly dependent on two ubiquitin-like conjugation systems. These are the ATG12-ATG5-ATG16L1 system and the LC3 conjugation system. These systems determine the site of lipidation of the LC3 protein by the ATG12-ATG5-ATG16L1 complex, which then cleaves LC3 to form LC3-I. This LC3-I is then combined with phosphatidylethanolamine to form LC3-II, a characteristic marker of autophagosomal membranes (Runwal et al., 2019; Mizushima, 2020; Boehi et al., 2021; Vujic et al., 2021).

The mTORC1 complex plays a critical role in regulating autophagy by inhibiting its initiation under nutrient-rich conditions (Ramaian Santhaseela and Jayavelu, 2021). Another key regulator is AMP-activated protein kinase (AMPK), which senses the energy status of cells and triggers autophagy during periods of energy depletion (Gelinas et al., 2018). In addition, the transcription factor EB (TFEB) modulates the transcription of autophagy and lysosomal genes, thereby maintaining autophagic balance (Dang and Back, 2021).

Autophagy is a response mechanism to various stressors, allowing the use of intracellular nutrients and the removal of damaged components (Schaefer and Dikic, 2021). However, it also has a dark side. Dysregulation of autophagy has been implicated in several pathologies, including neurodegeneration, heart disease, and cancer. Understanding how this critical, multifaceted process is regulated could potentially provide new avenues for therapeutic intervention in various disease contexts (Kroemer and White, 2010; Markaki and Tavernarakis, 2020).

In summary, macroautophagy, enabled by ATG proteins, is a primary form of autophagy. mTORC1 inhibits autophagy under conditions of nutrient abundance, whereas AMPK induces it under conditions of energy deprivation. While autophagy typically plays an adaptive role, its dysregulation can wreak havoc and lead to disease pathology.

## 4. Autophagy dysregulation in Alzheimer's disease

AD is associated with progressive neuronal deterioration leading to dementia. The key pathological features include extracellular amyloid plaques containing aggregated Aβ peptide and intracellular neurofibrillary tangles containing aggregated hyperphosphorylated tau protein (Patil et al., 2010; Ciccone et al., 2020). Autophagy, a form of cellular degradation involving lysosomal turnover of cytoplasmic material, is increasingly implicated in AD pathogenesis (Schaefer and Dikic, 2021).

Autophagy is one of the primary pathways for the degradation of aggregated proteins. In the context of AD, impaired autophagy-mediated clearance of Aβ and tau aggregates is likely to contribute to their accumulation. Aβ peptides are derived from amyloid precursor protein (APP), which is proteolytically cleaved by β- and γ-secretases. Accumulation of Aβ, particularly the neurotoxic oligomeric forms, results in synaptic dysfunction, neuronal loss, and cognitive decline (Festa et al., 2021; Ntsapi and Loos, 2021). Tau is a microtubule-stabilizing protein that resides within axonal microtubules under typical conditions. Hyperphosphorylation forces tau to disengage from microtubules and form neurotoxic oligomeric aggregates that disrupt neuronal transport (Gyparaki et al., 2021; Wang D. et al., 2021). Impaired autophagic clearance accelerates the accumulation of Aβ and tau aggregates, exacerbating neurodegeneration (Festa et al., 2021).

Research indicates that dysfunctional autophagy impairs clearance of Aβ and tau aggregates, promotes their accumulation, and increases neurotoxicity in AD models. APP transgenic mice with knockout of an autophagy gene, Beclin-1 or ATG7, show increased extracellular Aβ deposition and intracellular Aβ accumulation (Carosi et al., 2021; Festa et al., 2021; Wu et al., 2021). Pharmacological autophagy has been shown to reduce Aβ levels and ameliorate cognitive deficits in APP mice (Wu et al., 2021). The restoration of autophagy has been found to exert an ameliorative effect on tau pathology when tau mutation or aggregation inhibitors are used (Anglada-Huguet et al., 2021). Autophagy stimulation has been found to result in tau clearance in primary neurons, whereas autophagy inhibition results in the accumulation of tau aggregates (Chinnathambi and Gorantla, 2021). Therefore, it is likely that autophagy impairment contributes to Aβ and tau pathology in AD.

**Figure 2:**
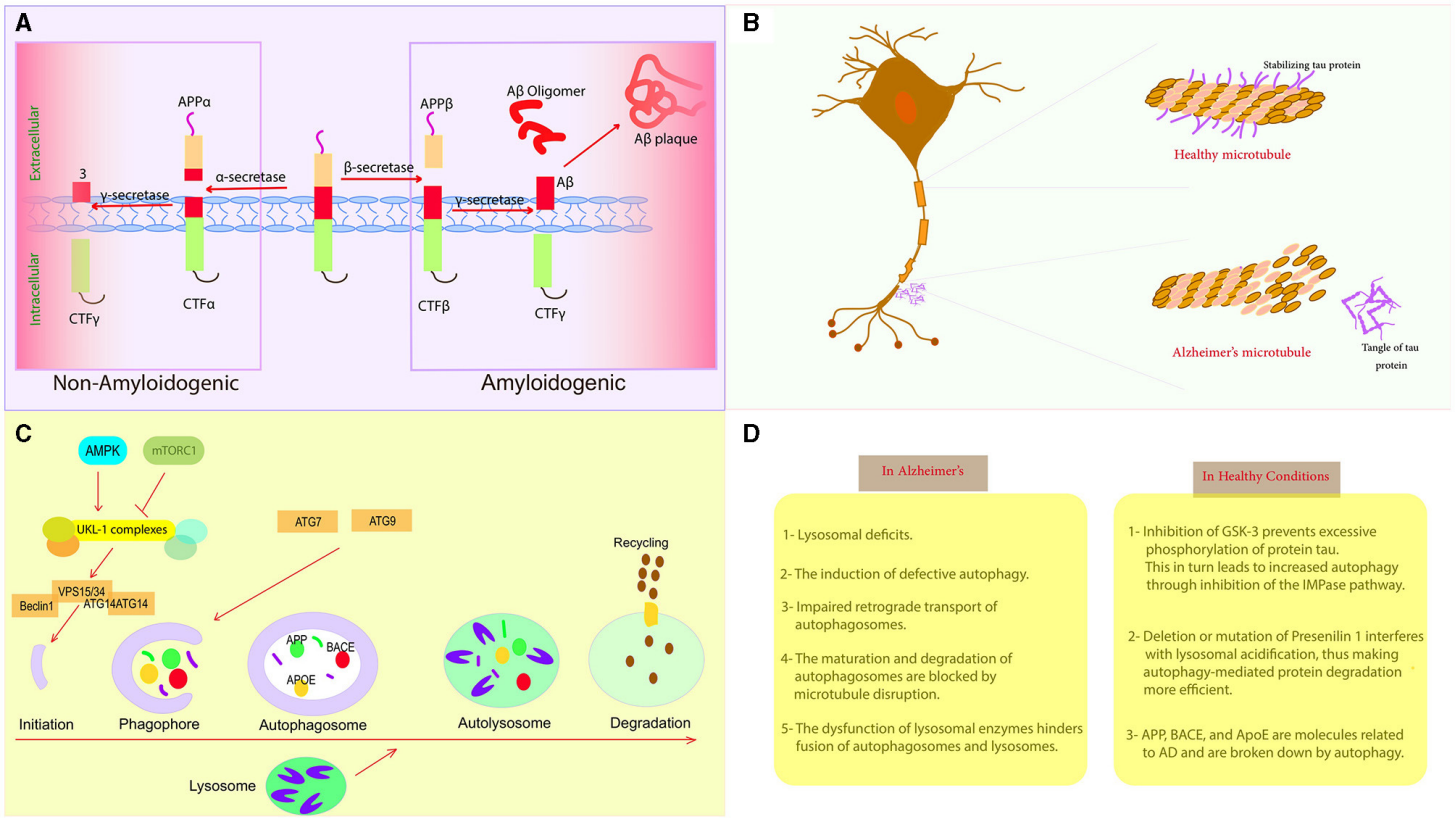
Autophagy and Alzheimer's disease. **(A)** AD development begins with the accumulation of abnormal Aβ peptides resulting from the proteolytic cleavage of APP by γ-secretase and β-secretase. **(B)** Tau is present in the axon of the neuron, but its hyperphosphorylated version is insoluble and unlikely to bind to microtubules, leading to axonal degeneration. **(C)** The primary processes that control different stages of autophagy. **(D)** Comparison between healthy conditions and the pathological conditions associated with AD.

Several lines of evidence support a role for defective autophagy in the pathogenesis of AD. Indicators of autophagy, such as LC3-II and Beclin-1, are reduced, while defective lysosomal proteolysis and autophagosome accumulation are evident in the brains of AD patients (Mani et al., 2021). Increased AD risk is associated with variations in autophagy gene polymorphisms (Lipinski et al., 2010). The accumulation of autophagy-promoting proteins is caused by Aβ and tau oligomers, which impair autophagy function, setting up a damaging cycle (Feng et al., 2020). Autophagy is disrupted by risk factors for AD, such as apo E4 and presenilin 1 mutations (Yang et al., 2019). Autophagy stimulation reduces Aβ and tau pathology and improves cognition in AD models (Qi et al., 2021). Thus, mitigating autophagy dysfunction represents an attractive strategy for AD treatment, potentially modifying the course of the disease. Table 2 provides an overview of the key genes associated with proteostasis, while Figure 2 graphically illustrates the role of autophagy processes in the context of AD. In conclusion, impaired autophagic clearance of Aβ and tau aggregates likely contributes to their accumulation in AD. Enhancing autophagy helps to clear toxic protein aggregates and prevent neurodegeneration, providing strong evidence that autophagy dysfunction is an important factor in AD pathogenesis. Targeting the autophagy pathway may provide therapeutic opportunities for AD.

**Table 2:** Genes associated with proteostasis.

**Gene**	**Function in proteostasis**	**Mutations**	**References**
APP	APP is involved in the metabolism, distribution, and formation of proteins.	Alterations in APP can lead to the accumulation of amyloid plaques.	Lloyd et al., 2020; Lin et al., 2021
PSEN1, PSEN2	PSEN1 and PSEN2, elements of the γ-secretase complex, are involved in the modulation of APP and the generation of Aβ peptides.	Any genetic alteration within PSEN1 and PSEN2 has the potential to increase the production and accumulation of Aβ.	Pimenova and Goate, 2020; Liu et al., 2021
MAPT	MAPT plays a critical role in strengthening the stability and organization of microtubules. In AD, there is increased accumulation resulting in neurofibrillary tangles.	Genetic alterations in MAPT could cause abnormal aggregation of tau and consequently abnormal tangles.	Venkatramani and Panda, 2019; Leveille et al., 2021
BACE1	BACE1 is an enzyme that plays a crucial role in the manipulation and creation of Aβ peptides.	Genetic alterations in BACE1 can influence APP cleavage.	Tambini et al., 2019; Zhuravleva et al., 2021
TREM2	TREM2 is a critical component of microglial activity and the body's immune response. It has been suggested that alterations or mutations in TREM2 may increase predisposition to develop late-onset AD.	Mutations in TREM2 are linked with a heightened likelihood of AD and disruption in proteostasis.	Steiner et al., 2020; Sayed et al., 2021

APP, amyloid precursor protein; PSEN1, presenilin 1; PSEN2, presenilin 2; MAPT, microtubule-associated protein tau; BACE1, beta-site amyloid precursor protein (APP)-cleaving enzyme 1; TREM2, triggering receptor expressed on myeloid cells 2.

## 5. Autophagy in animal models of AD

In animal models of AD, the introduction of bone marrow-derived mesenchymal stem cells (BMMSCs) has been shown to induce autophagy and reduce apoptosis, resulting in improvement of memory and cognitive deficits (Qin et al., 2022). The induction of autophagy is supported by the observed expression of signaling molecules, including Beclin-1, Atg5, LC3-II, and mTOR (Qin et al., 2022). Research beyond BMMSC transplantation has also investigated the function of autophagy in AD using various animal models. In one case, an APP transgenic mouse model with deletion of Beclin-1 showed a disruption in the basic level of autophagy, leading to escalation of the accumulation of Aβ in the cell (Liu and Li, 2019). This suggests that maintaining the flow of autophagy may be essential to prevent Aβ accumulation and subsequent neuronal death (Liu and Li, 2019). In addition, recent evidence suggests that specific perturbations of non-canonical autophagy in microglia may reduce the ability to clear β-amyloid, leading to progressive neurodegeneration in a mouse model of AD. These observations are consistent with previous findings in mice lacking autophagy-related gene 7 (atg7) (Wang Z. et al., 2022). Intriguingly, some research has suggested that boosting autophagy may have protective effects on the nervous system. For example, administration of rapamycin, a known mTOR inhibitor that triggers autophagy, has been shown to reduce Aβ levels and improve cognitive function in mouse models of AD (Liu and Li, 2019). In conclusion, these studies highlight the potential therapeutic role of autophagy modulation in AD. However, further research is needed to fully understand the complex interplay between autophagy and other cellular processes in the context of AD. The use of animal models will continue to be essential in this endeavor.

## 6. Autophagic biomarkers in AD

Recent research has aimed to explore the deep relationship between autophagy and AD, with the aim of discovering possible autophagy-related biomarkers for Alzheimer's disease. Researchers aim to do this by identifying critical autophagy genes that show striking differences in expression and investigating the potential roles of these genes (Li et al., 2023). One study has discovered ten DEAGs that play a role in the progression of AD. These include nine genes with increased activity (CAPNS1, GAPDH, IKBKB, LAMP1, LAMP2, MAPK1, PRKCD, RAB24, and RAF1) and one with decreased activity (CASP1). Correlational analysis has suggested possible links between these key DEAGs (Li et al., 2023). In addition to these genetic biomarkers, other studies have identified dysfunctional autophagic processes in the brains of Alzheimer's patients. For example, the accumulation of immature autophagic vacuoles (AVs) in dystrophic neurites in the brains of AD patients suggests that the autophagic process is disrupted (Liu and Li, 2019). In addition, presenilin has been proposed to play a necessary role in lysosomal calcium storage and release; without proper presenilin function, cells exhibit defective endosomal–lysosomal fusion, accompanied by the accumulation of endosomes and autophagosomes, and severely deficient autophagy (Orr and Oddo, 2013). The accumulation of Aβ protein fragments outside neurons, known as “Aβ plaques”, and the accumulation of an altered variant of the tau protein inside neurons, known as “tau tangles”, are two notable brain changes associated with AD4 (Wang Y. Y. et al., 2021). These pathological features could also be used as potential indicators of the presence of AD. In summary, these studies highlight the prospective importance of autophagy-related biomarkers for the detection and treatment of AD. However, further studies are essential to fully unravel the intricate relationship between autophagy and other cellular functions within the AD spectrum.

## 7. Crosstalk between autophagy and other cellular pathways

Recent research suggests that autophagy is involved in several other cellular pathways in AD, such as the ubiquitin–proteasome system (UPS), the endoplasmic reticulum (ER) stress response, and the unfolded protein response (UPR) (Kocaturk and Gozuacik, 2018). The UPS functions to degrade misfolded proteins and maintain protein balance in the cell. Recent research has shown that autophagy and the UPS undergo a complex interaction in the context of AD (Kocaturk and Gozuacik, 2018). One study found that the inhibition of autophagy led to the accumulation of ubiquitinated proteins and the activation of the UPS in AD animal models. The study also found that inhibition of the UPS led to the accumulation of autophagic vacuoles and the activation of autophagy in these models (Ke, 2017). These findings suggest that autophagy and the UPS interact in AD and that the regulation of these pathways may be a potential therapeutic target for the treatment of the disease. The ER stress response and the UPR are cellular pathways that respond to misfolded proteins and regulate protein balance. Recent research has shown that autophagy interacts with these pathways in AD (Chipurupalli et al., 2021). One study showed that when autophagy was inhibited, the ER stress response and the UPR were subsequently triggered in models of AD (Montibeller and de Belleroche, 2018). In addition, when autophagy was activated, there was a decrease in ER stress and the UPR in these models. These results suggest that autophagy interacts with the ER stress response and the UPR in AD, and that targeting these pathways could also be a potential therapeutic focus for managing the disease (Tooze et al., 2017). In addition to the UPS, the ER stress response, and the UPR, recent studies have shown that autophagy interacts with other cellular pathways in AD, including the immune response and the mitochondrial quality control system. One study found that activation of autophagy led to a reduction in neuroinflammation and an improvement in cognitive function in animal models of AD (Wang G. et al., 2019). The findings also indicated that the induction of autophagy led to an improvement in mitochondrial functionality and a reduction in oxidative stress in these models. These findings suggest that autophagy is involved in the immune response and the system that monitors mitochondrial quality in AD (Lee et al., 2012; Hyttinen et al., 2021). Control of these pathways may offer potential therapeutic strategies to combat the disease. In summary, autophagy dysregulation is a key feature of AD, and recent studies have shown that autophagy interacts with other cellular pathways in the disease, including the UPS, ER stress response, UPR, immune response, and mitochondrial quality control system. These findings suggest that regulation of these pathways may be potential therapeutic targets for the treatment of AD. Further research is needed to fully understand the crosstalk between autophagy and other cellular pathways in AD and to develop effective therapeutic strategies.

## 8. Therapeutic potential of modulating autophagy

Modulation of autophagy already offers a promising avenue for therapy given the contribution of autophagy dysfunction to AD pathology (Kaushik et al., 2021; Eisenberg et al., 2022).

Therapeutic approaches to modulation ofautophagy include a variety of strategies, including dietary control, pharmacological methods, and genetic therapies.

Of note is the potential of caloric restriction to induce autophagy by inhibiting mTOR signaling, a prominent inhibitor of autophagy. Stimulation of autophagy leads to significant improvements in cognitive function and reduced levels of amyloid-beta plaques and phosphorylated tau in mouse models of Alzheimer's under 30% dietary restriction. However, long-term dietary abstinence seems largely infeasible for AD patients (Ferreira-Marques et al., 2021; Muller et al., 2021). The feasibility tends toward clinical relevance in the case of the use of pharmacological inducers of autophagy such as mTOR inhibitors and lithium. Notably, the mTOR inhibitor rapamycin has been found to increase autophagy levels, reduce amyloid-beta and phosphorylated tau levels, and improve cognition in AD mouse models (Yang and Zhang, 2020). Lithium's similar enhancement of autophagy and attenuation of amyloid-beta and tau pathology enhances its therapeutic potential for AD (Morris and Berk, 2016; Uddin et al., 2021).

Evidence suggests that autophagy, along with its non-canonical processes such as LC3-associated phagocytosis (LAP) and LC3-associated non-canonical autophagy-dependent necroptosis (LANDO), plays a significant role in the development of AD (Hooper et al., 2021; Magne and Green, 2022). Recent evidence suggests an emerging role of LANDO in cytokine receptor signaling and innate immunity (Magne and Green, 2022). Research study by Martinez-Vicente et al. has shown that LC3-associated phagocytosis (LAP) plays an important role in Aβ plaque clearance. When LAP is inhibited in microglia, Aβ plaque clearance is reduced and inflammation is increased (Martinez-Vicente et al., 2010). In addition, their research in a mouse model of AD showed increased LAP activity in microglia located around Aβ plaques. This further supports the critical role of LAP in reducing Aβ in AD (Martinez-Vicente et al., 2010). A study led by Zhang et al. has presented evidence that triggering LANDO leads to nerve cell death and cognitive decline. Conversely, in a mouse model of AD, blocking LANDO was found to lead to reduced nerve cell death and improved cognitive performance. This raises the possibility that LANDO may be a potential candidate for AD therapy (Wang J. et al., 2019). Microglia, the resident immune cells of the CNS, play a crucial role in the pathogenesis of age-related neurodegenerative diseases (NDDs). Emerging evidence suggests that microglia coordinate the inflammatory responses in the CNS and contribute to the progression of NDDs (Lin et al., 2022). Microglial autophagy is involved in the regulation of neuronal homeostasis and the process of neuroinflammation. Dysregulation of microglial autophagy has been implicated in the pathogenesis of NDDs, including AD, PD, and HD (Navone et al., 2015; Lin et al., 2022). These findings suggest that the regulation of microglia autophagy and mitophagy may be a potential therapeutic target for the treatment of NDDs.

Gene therapy strategies such as overexpression of autophagy-related proteins, including Beclin-1, stimulate autophagy to reduce AD pathology in animal models (Pickford et al., 2008). In addition, small molecules capable of activating the autophagy function of Beclin-1 show protective effects in AD models (Seo et al., 2014). Thus, these early promising results demonstrate the potential of genetic and pharmacological approaches to enhance autophagy in AD therapy.

However, translating autophagy modulation to actual patients presents several challenges. The complex and dynamic nature of autophagy is closely intertwined with numerous cellular pathways, which may lead to physiological perturbations following non-selective autophagy induction (Ariosa et al., 2021; Manea and Ray, 2021). Unexpected cytotoxic or tumorigenic effects remain a concern with long-term use, as autophagy can oscillate between promoting cell survival and death (Zaarour et al., 2021).

Pharmacological agents are also not without problems—poor target specificity and minimal permeability of the blood–brain barrier, coupled with systemic side effects during long-term use, nullify their efficacy (Sitta, 2018; Zaarour et al., 2021). Therefore, the successful development of autophagy-based treatment strategies for neurodegeneration will require improvements in central nervous system (CNS) delivery, optimization of treatment duration and timing, and enhancement of cell and tissue specificity (Ajoolabady et al., 2021).

While preliminary results seem promising, most studies have examined short-term treatments in younger transgenic mouse models of AD (Oblak et al., 2021). The resulting long-term effects on cognition and pathology in older animal models, which better reflect AD progression, are areas that require further investigation (Munoz-Moreno et al., 2020). Future research should also examine the effects on neuroinflammation and synapse loss, which are key aspects of AD pathogenesis.

In summary, interventions aimed at restoring autophagy have been shown to ameliorate AD pathology and symptoms at the preclinical stage. However, successful translation to the clinic requires careful consideration of a variety of factors: unintended effects, disease stage, duration of treatment, and age-related changes in autophagy. Only then can autophagy be safely and effectively modulated for AD therapy. Table 3 illustrates a selection of ongoing clinical trials and drugs targeting proteostasis pathways for the treatment of AD.

**Table 3:** Current clinical trials and drugs targeting proteostasis pathways to treat AD.

**Drug/intervention**	**Mechanism**	**Phase of clinical trial**	**References**
ALZT-OP1a	Vaccine targeting misfolded Aβ oligomers	Phase 3	Jeremic et al., 2021
Aducanumab	Monoclonal antibody targeting Aβ aggregates	FDA approved	Nistico and Borg, 2021
CAD106	Vaccine inducing antibodies against Aβ	Phase 2	Vandenberghe et al., 2017
VERubecestat	BACE1 inhibitor to reduce Aβ production	Phase 2/3 (terminated)	Chris Min et al., 2019
Tau antisense oligonucleotides	Reduction in tau production	Phase 1	DeVos et al., 2017
Nilotinib	Tyrosine kinase inhibitor that targets tau aggregation	Phase 2	Nobili et al., 2023
Rapamycin	mTOR inhibitor that enhances autophagy	Phase 2	Subramanian et al., 2022
NLY01	GLP-1 receptor agonist to promote neurogenesis	Phase 2	Park et al., 2021

## 9. Future directions and research gaps

A number of novel methodologies offer potential advances in our understanding of autophagy and proteostasis in AD. For example, the advent of super-resolution fluorescence microscopy has been instrumental in the study of autophagic structures at the nanoscale, allowing for a more comprehensive understanding of autophagosome formation, cargo sequestration, and lysosome fusion (Karanasios, 2019; Zheng et al., 2021). In addition, innovative live cell imaging methods provide the ability to monitor fluorescently tagged autophagy proteins together with selective autophagy substrates (Perez-Leal et al., 2021). Equipped with high-content screening platforms, it is becoming increasingly possible to identify novel compounds capable of modulating autophagy by allowing phenotype-based screening in neuronal models (Suenaga et al., 2021). Using the sophisticated techniques of single-cell transcriptomic profiling, the cell state-specific roles of autophagy genes in AD models can be more clearly delineated (Hu et al., 2017). These state-of-the-art methods are enabling an unprecedented level of understanding of autophagic mechanisms in the context of AD pathogenesis (Mijaljica, 2010).

Despite these advances, there are significant gaps in our knowledge of autophagy and proteostasis in AD. Determining the specific molecular pathways that link Aβ and tau pathologies to altered neuronal autophagy remains a critical task (Zhao et al., 2020). It is also unclear which aggregated forms exert the greatest toxicity and whether their accumulation is a cause or a symptom of autophagy dysfunction. The role of autophagy in brain cells such as microglia, which have been implicated in AD, remains largely unexplored (Pomilio et al., 2020). Understanding shifts in proteostasis networks during the early prodromal stages, before plaques and tangles accumulate, is critical. The question of why certain neuronal populations are more vulnerable than others also remains unanswered. The paucity of longitudinal patient studies monitoring cognitive decline along with cerebrospinal fluid (CSF) and imaging markers of autophagy calls for urgent attention (Mufson et al., 2016). Sophisticated investigations of these critical issues are expected to provide key insights into the pathogenesis of AD.

The understanding that diminished autophagy plays a role in AD progression has raised the potential of targeting autophagy with therapies. Drugs that induce the onset of autophagy have shown encouraging signs in AD models (Jogalekar et al., 2021; Kaushik et al., 2021). In addition, manipulation of late-stage autophagy may also prove beneficial. By controlling lysosomal dysfunction, proteostatic capacity can be significantly enhanced in AD models (Xu et al., 2022). While gene therapies aimed at increasing autophagy have shown efficacy in preclinical studies, most strategies aimed at modulating autophagy have yet to reach the clinic (Long and McWilliams, 2020). Predicting patient response, managing unanticipated side effects, and ensuring adequate CNS delivery of autophagy-modulating agents remain challenges. However, the identification of autophagy-related biomarkers may provide potential patient segmentation for targeted therapies (Pani and Keefe, 2019). Ultimately, a comprehensive approach to proteostasis therapies that simultaneously enhances autophagy, lysosomal function, and proteasomal activity may provide the most effective treatment for AD (Njomen and Tepe, 2019). Overall, modulation of neuronal autophagy and proteostasis represents a promising avenue for the development of individualized AD therapeutics.

## 10. Conclusion

Disturbances in proteostasis and autophagy are major contributors to AD. These perturbations result in the accumulation of misfolded proteins, including amyloid-beta and tau, due to an imbalance in protein production and clearance. The critical degradation function of autophagy is compromized, leading to the accumulation of toxic protein aggregates. Research has shown that molecular chaperones and the ubiquitin–proteasome system play an important role in managing proteostasis and influencing the aforementioned pathologies. Several autophagy pathways are critical for clearance of protein aggregates and dysfunctional mitochondria in AD. Impaired proteostasis and autophagy lead to oxidative stress, synaptic dysfunction, and neuronal death, accelerating AD neurodegeneration. A deeper understanding of these intricate cellular activities will be instrumental in identifying new drug targets and developing effective disease-modifying treatments. Efforts to repair proteostasis and enhance autophagy are promising therapeutic strategies for AD that require continued exploration to potentially move findings from the research stage to practical application. Ultimately, substantial evidence suggests that proteostasis networks and autophagy are critical in the development of AD and are promising areas for future treatments.

## Author contributions

AN: Visualization, Writing—review & editing. HB: Visualization, Writing—original draft. FK-K: Supervision, Writing—review & editing.
